# Cultural consensus and intracultural diversity in ethnotaxonomy: lessons from a fishing community in Northeast Brazil

**DOI:** 10.1186/s13002-022-00522-y

**Published:** 2022-03-27

**Authors:** Vítor Renck, Deborah M. G. Apgaua, David Y. P. Tng, Paride Bollettin, David Ludwig, Charbel N. El-Hani

**Affiliations:** 1 National Institute of Science and Technology in Interdisciplinary and Transdisciplinary Studies in Ecology and Evolution (INCT IN-TREE), Federal University of Bahia, Salvador, Brazil; 2 Institute of Biology, Federal University of Bahia (UFBA), Brazil, R. Barão de Jeremoabo, 668, Salvador, BA 840170-115 Brazil; 3 Centre for Rainforest Studies, The School for Field Studies, Yungaburra, QLD 4884 Australia; 4 Centre for Tropical, Environmental and Sustainability Sciences, College of Science and Engineering, James Cook University, 14-88 McGregor Rd, Smithfield, QLD 4878 Australia; 5 Department of Anthropology, Faculty of Science, Masaryk University, Brno, Czech Republic; 6 Knowledge, Technology, and Innovation Group, Wageningen University and Research, Wageningen, The Netherlands

**Keywords:** Artisanal fishers, Ethnobiology, Ethnozoology, Indigenous and local knowledge, Free list, Triad task

## Abstract

**Background:**

Traditional fishing communities are strongholds of ethnobiological knowledge but establishing to what degree they harbor cultural consensus about different aspects of this knowledge has been a challenge in many ethnobiological studies.

**Methods:**

We conducted an ethnobiological study in an artisanal fishing community in northeast Brazil, where we interviewed 91 community members (49 men and 42 women) with different type of activities (fishers and non-fishers), in order to obtain free lists and salience indices of the fish they know. To establish whether there is cultural consensus in their traditional knowledge on fish, we engaged a smaller subset of 45 participants in triad tasks where they chose the most different fish out of 30 triads. We used the similarity matrices generated from the task results to detect if there is cultural consensus in the way fish were classified by them.

**Results:**

The findings show how large is the community’s knowledge of fish, with 197 ethnospecies registered, of which 33 species were detected as salient or important to the community. In general, men cited more fish than women. We also found that there was no cultural consensus in the ways fish were classified.

**Conclusions:**

Both free-listing and triad task methods revealed little cultural consensus in the way knowledge is structured and how fish were classified by community members. Our results suggest that it is prudent not to make assumptions that a given local community has a single cultural consensus model in classifying the organisms in their environment.

**Supplementary Information:**

The online version contains supplementary material available at 10.1186/s13002-022-00522-y.

## Introduction

Human societies have always built ways of making sense of the biological diversity surrounding them, for instance, by grouping organisms by similarity or separating them by difference [1, 2]. These categorization processes are culturally influenced and organized in different taxonomic structures [3, 4]. One of the most striking observations in ethnobiology is the common occurrence of agreement both within and across cultures in the categorization of plants and animals [5–7]. However, ethnobiologists have also documented many differences in intra- and intercultural categorization, and other factors such as age, social role, and religion may influence categorizations of nature [8–10].

In fishing communities all around the world, fishers have developed their own classification systems to name and organize the fish they use [1]. For example, in Rio de Janeiro, Brazil, the fishers from Sepetiba Bay categorize fish by morphological, ecological, economical value, and meat quality [11]. At another location in Rio de Janeiro, called Piratininga, fishers base their classification on how fish behave [12]. Begossi and Garavello [13] observed that the criteria used by the Tocantins River fishers, in the Brazilian states of Maranhão and Tocantins, are mainly morphological. The indigenous and fishing community of Piaroa, in Colombia, classifies fish and other animals based on food taboos and religious elements [14].

Ross et al. [15] criticized the simple approach of reporting species lists of organisms in ethnoscientific research, raising the question of whose knowledge is being reported. More specifically, Ross et al. [15, p. 270] assert that, “in most descriptions of folk-knowledge systems, it is not clear whether the reported knowledge is held by every individual or even by any single individual in a community. Most often, neither is the case; instead, the data represent an artificial collage of knowledge bits reported by individuals and put together by the author in a systemic fashion”.

In order to understand the knowledge available in a community, and how it is shared among its members, scholars have adopted the approach of inquiring into cultural consensus (or its lack) within communities. Romney et al. [16] proposed the so-called *cultural consensus model* (CCM), which is a method for computing levels of agreement and disagreement in the structure and distribution of information within and across populations. The model assumes that widely-shared information is reflected in a high level of agreement, or “cultural consensus” among individuals [7]. Some examples of ethnobiological researches that used this methodology are Ross et al. [15], who studied plant categorization by the Tzotzil Maya from Zinacantán (Highlands of Chiapas, Mexico), Medin et al. [17], who studied the categorization of trees by tree experts in the Chicago region (USA) and Medin et al. [7], who studied the categorization of freshwater fish by Native American and majority-culture fish experts from the north central Wisconsin (USA). While the two latter document intercultural variation, the former analyze intracultural variation in the Tzotzil Maya biological knowledge.

The intracultural variation of indigenous and local knowledge (ILK) is patterned following sociodemographic characteristics of community members, geographic characteristics as well as domains of knowledge [18]. Reasons for the intracultural variation of ILK are suggested to include gender [19–21], age [22–24], levels of expertise [25], distribution of work [20], among others. Analyzing intracultural variation of ILK enables scholars to raise hypotheses about the social organization in a culture, gives indications of persistence or loss of ILK and thereby help to identify the conditions for its thriving and vanishing [18].

The need for a better understanding of Indigenous and local biological knowledge in many parts of the world as well as its growing disappearance is of great concern to ethnobiologists [46]. The loss of local biological knowledge is often attributed to globalization, environmental degradation, and disruptive changes in social and economic systems [see [26–30]. Even though indigenous and local knowledge is being lost at an alarming rate [26, 28], many communities maintain local knowledge and practices despite the threats impacting them. A number of artisanal fishing communities in Brazil provide notable examples [31, 32].

Over the last five years, we have built a trust relationship and engaged in a research-action project to build up dialogue with members of two artisanal fishing communities in the Itapicuru estuary, in Northeast Brazil, and engaged in documenting and preserving their ethnobiological knowledge [e.g. [21, 33]. The present study is part of a project that is interdisciplinary, involving a team of researchers from different academic areas and from different institutions seeking to build an integrated body of knowledge, and transdisciplinary, aiming to produce an effective and symmetrical dialogue with the non-academic knowledge, held by other social actors, such as fishers.

During this time, we have been performing mixed-methods studies in the areas of intercultural education, biodiversity conservation, and ethnobiology. One aspect of the local artisanal fishers’ ethnobiological knowledge still lacking documentation is their knowledge of fish, which is the focus of this study. In addition to a lack of understanding about whether and to what extent there is cultural consensus among the fishers and other community members, how fish knowledge is patterned by gender and type of activity (fishers or non-fishers) is also in need of inquiry.

Therefore, the overarching aim of this study is to understand how local knowledge in an artisanal fishing community situated in Northeast Brazil is distributed among its members. More specifically, we aim to: (1) document the most culturally salient fish (ethno)species (locally considered as being of high importance) in the community; (2) determine if there is a consensual cultural model regarding fish ethnotaxonomy; and (3) examine how gender and type of activity influence differences in intracultural knowledge composition within the fishing community.

## Methods

### Study area

The fishing village of Siribinha (11°48′49"S, 37°36′38"W) is part of the municipality of Conde and is located in the Itapicuru estuary in Bahia, in northeast Brazil. The area consists of freshwater alluvial wetlands, mangroves, beach vegetation, and shrubby thicket-like forests (locally known as *restingas*) growing on sand dunes. Coconut plantations and cattle ranches also make up part of the land use tenure of the region [21].

Siribinha is a community of artisanal fishers comprising ca. 500 inhabitants. The community was relatively isolated up to the 1990s, since prior to that there was no road connecting it to nearby villages and cities. Despite the emergence of small-scale tourism starting from the mid-1990s, Siribinha is still predominantly a fishing community, where fishing and shellfish gathering constitute the main economic activity of the majority of the community members.

Artisanal fishing in the north coast of Bahia is characterized by family work, where members of the family are variously involved in the activity of catching and processing the catch, especially shellfish. In Siribinha, as well as in other Brazilian fishing communities, fishing is typically a male activity, while shellfishing, which comprises the activity of gathering mollusks and crustaceans, is carried out primarily by women and children [1].

Most of the information on the community provided here results from our own interview data and participant observation in the larger project in which the present study is included.

### Data collection

Throughout the paper, we indicate the community members by the initial letter of their names followed by a dot and their age (e.g. E.68), for confidentiality reasons. The Portuguese transcripts were translated by the first author and the translation was revised by the other authors. In the quotes from community members’ interviews, pauses are indicated with a slash (/), and the end of a speech turn, with a period (.). The transcripts are shown in italics and, if we need to comment or add something, this is done using parentheses, without italics. For each transcript included in the paper, we provide the Portuguese original excerpts in the Additional File 1. To carry out the research reported here, we combined two methods: free listing and triad tasks.

### Free listing

For our specific purpose of understanding fish ethnotaxonomy, we performed a free listing task to determine the most salient species of fish. Free listing interviews were performed either in their houses, during door-to-door visits, or in the shared social spaces in the village. Most interviews happened in their free time, i.e., during the day when they were at home or sitting on their porches, but some of them were also done when they were repairing their nets or landing fish. Their consent to be interviewed was obtained in audio recordings after stating the terms of an Informed Consent Form.

The free listing was carried out in April 2018 with 91 community members (approx. 20% of the community), comprising a total of 49 men and 42 women, aged 18–89 years old. The interviewees consisted of 38 fishermen and 25 fisherwomen (shellfish gatherers), but also another 11 male and 17 female community members dedicated to other activities (students, teachers, traders, accommodation providers, among others). We considered fishermen or fisherwomen those who engaged in fishing/shellfish gathering activities ≥ 3 days a week or, in the case of retirees, if they had engaged in these activities at such an intensity before retiring.

Each participant was asked “What fish do you know?” We then let the interviewees speak freely so as not to influence their train of thought, and we noted the cited fish in the order they were mentioned. In instances where interviewees cited ethnogenera of fish such as *arraia* (stingray), *bagre* (catfish), *cação* (small shark), *pescada* (hake), and *robalo* (snook), we sought further clarification by asking later if there is more than one type of each of them. If that was the case, these would be annotated just after each ethnogenus mentioned.

### Triad tasks

To understand how members of the community categorize fish and to what extent the categories are shared across the community, we carried out triad tasks (or triad tests) [15, 34]. The triad task allows us to derive a consensual cultural model without assuming that such a model exists beforehand [15]. For this purpose, we randomly selected 45 people that took part in the free list task (15 fishermen, 15 shellfish gatherers/fisherwomen, and 15 other community members) and solicited their participation on the triad tasks. The triad tasks were conducted between October 2018 and January 2019.

During the triad task, a series of 10 sets of three photographs (a triad) of fish were presented to each participant. To generate the triad tasks, we randomly selected ten ethnospecies of fish (Table 1) among the 33 most salient to the community, according to the findings from the free listing interviews (see section “Free Lists and Salience Indices'' in the Results). They were then asked which ethnospecies was the most different among the three shown in the photographs. It was then assumed that the two other ethnospecies were being considered more similar to each other by the interviewee. If the participant had difficulty with a specific triad, that triad was postponed until the end of the task. If the participant.

**Table 1:** Ethnospecies of fish selected for the triad tasks (in alphabetical order) in Siribinha, northeast Brazil

Ethnospecies	Academic scientific species (family)
Bagre fidalgo	*Bagre bagre* (Ariidae)
Carapeba	*Eugerres brasilianus* (Gerreidae)
Cavala	*Scomberomorus cavalla* (Scombridae)
Corvina	*Micropogonias furnieri* (Sciaenidae)
Curimã	*Mugil liza* (Mugilidae)
Pescada amarela	*Cynoscion acoupa* (Sciaenidae)
Pescada branca	*Cynoscion leiarchus* (Sciaenidae)
Robalo branco	*Centropomus parallelus* (Centropomidae)
Tainha	*Mugil curema* (Mugilidae)
Xaréu	*Caranx hippos* (Carangidae)

still could not provide an answer after this second round of questioning, he or she was asked whether the difficulty of making the requested judgment was due to the ethnospecies being very similar or very different. For each attempt, participants could choose an item, therefore, as “different” (codes 1–3/1—item on the left/2—item on the middle/3—item on the right), “very different” (code 0) or “very similar” (code 4). We followed this procedure to avoid situations where participants felt forced to produce an answer, thereby choosing items randomly and biasing the data [15].

The triad task was performed with a Lambda 2 design [34, 35], which means that each pair of ethnospecies was compared exactly twice. Using 10 ethnospecies, this design generates 30 triads, a number that was deemed manageable for the triad tasks. A higher number of ethnospecies was used in pilot interviews. However, the interviews were too long (lasting approximately 50 min), tiring the interviewees and thereby compromising data quality.

The photographs of fish were taken with the same camera approximation to ensure that fish body size proportions were maintained. All the photographs used in the triad task are presented in the Additional file 1: Table S1.

To obtain an idea of how the participants classified the fish they saw in the photographs, we also asked them at the end of the triad tasks which criteria they used to differentiate one fish from the others.

### Data analysis

During the free listing tasks, we observed that some interviewees provided two or more different names or synonyms for some fish. By analyzing the whole set of interviews, we concluded that these different names referred to the same ethnospecies. For example, small phonetic differences were common, like *arraia jamanta* and *arraia jalamanta* (*Mobula *sp*.*) or *bagre upemba* and *bagre urupemba* (unidentified species). In some other cases, different fish names were also recognized by the interviewees as referring to one single ethnospecies, but at different ontogenetic phases, like *saúna* (smaller) and *tainha* (bigger) (*Mugil curema*) or *pescada amarela* (smaller) and *pescada selvagem* (bigger) (*Cynoscion acoupa*). Therefore, when running the analyses, we selected the most frequently used name by the community for each fish to which there were synonyms, in order to avoid artificially inflating the number of fish mentioned, thus biasing the salience estimation. The scientific species names for the ethnospecies and ethnogenera were provided by a fisheries specialist, Dr. José Amorim dos Reis Filho, based on the ethnospecies’ names. Dr. Dos Reis Filho investigates fishers' practices and has extensive knowledge of the species named by fishers in the study region. A rarefied ethnospecies-interviewee curve (Additional file 1: Figure S1) indicated that the number of participants engaged to generate our free lists was adequate, as the number of ethnospecies listed approached an asymptote.

To determine whether the gender and activity of the interviewees had any influence on the number of ethnospecies (or ethnogenera) cited, we performed two-way ANOVAs with gender (male, female) and activity (fishers, other activities) as factors (*α* = 0.05).

We calculated the Salience Index (S) of each fish ethnospecies following Chaves et al. [36], using the formula: S = Σ((L–Rj + 1)/L))/N, where L is the length of a list, Rj is the rank of item j in the list, and N is the number of lists in the sample. The index takes into account not only the frequency of occurrence of each item, but also the order in which they were mentioned in the interviews [36]. In cases where an interviewee mentioned an ethnogenus, for instance, *cação* (small shark), as the first item but mentioned more specific ethnospecies later, we substituted the ethnogenus for the mentioned ethnospecies. However, in some cases in which the interviewee did not provide any ethnospecies for an ethnogenus when questioned at the end of interview, we maintained the ethnogenus as he or she listed.

The Salience Index of each ethnospecies cited is calculated by the probability of occurrence of these values in a null scenario [36], and varies between 0 and 1, which denote ethnospecies with extremely low or high salience, respectively. The *p* values of salience show the probability that the salient values occur in a null scenario, calculated from simulated populations with similar characteristics to the real one, using Monte Carlo techniques [36]. Following Chaves et al. [36], we accepted a threshold *p* value < 0.05 to denote significance. Using this methodology, it is possible to establish a cut-off point in a free list and select only the most salient items, i.e., the ones showing high salience index and *p* value < 0.05.

To visualize how fish knowledge varies between interviewees (i.e. how their knowledge composition varies), we used a non-metric multidimensional ordination to summarize all the ethnospecies citations by the interviewees. The fish citations were coded as presence-absence data and used to compute a Bray–Curtis dissimilarity matrix [37]. In the ordination graph, interviewees were represented using different markers to denote different genders and activities [male—fisher, male—other (non-fisher), female—fisher, female—other (non-fisher)]. A permutational multivariate analysis of variation (PERMANOVA; α = 0.05) was run to determine if the composition of knowledge differed between the four groups. A Bonferroni correction was applied in order to reduce the chances of obtaining false-positive results.

To analyze the triad task data, we used the Anthropac 4.98 software [38]. Anthropac generates an “aggregate proximity matrix”, which is a similarity matrix showing the percentage of times each pair of ethnospecies were considered more similar within a triad (agreement = matched responses/30). Using these similarity matrices generated by Anthropac, we performed a non-metric multidimensional scaling ordination to determine how the participants were categorizing the fish and to visualize the degree of similarity between them according to the participants.

For each triad, Anthropac also provides the agreement rate, which is the proportion of triads in which each participant agreed with the modal response (i.e., that pair of fish which most participants considered the most similar within each triad). Using a principal components analysis (PCA) on the interviewee vs. interviewee matrix also enabled us to determine the presence of a cultural consensus model [16]. Widely shared information would be reflected in a “cultural consensus” or high agreement among individuals. To achieve this, each interviewee’s fish–distance matrix was correlated with that of every other interviewee, yielding a 45 × 45 matrix in which entries correspond to the observed agreement among interviewees on pairwise fish distances. A PCA was then performed on the inter-participant correlation matrix. Following Romney et al. [16], we deemed that a strong group consensus existed if (1) the ratio between the first and second factor eigenvalue was high, (2) the first eigenvalue accounted for a large portion of the variance, and (3) all individual first-factor scores were positive and relatively high. If these criteria are met, the structure of the agreement can be explained by a single factor solution, namely, the consensual model; otherwise, we may reach reliable conclusions about inter-participant differences [15].

We also carried out PERMANOVAs on the interviewee fish-distance matrices to determine if gender, activity and their interactions had any bearing on fish ethnotaxonomic classification. All analyses were performed in R (R Core Team 2017). The R script for calculating Salience indices was provided by L. Chaves upon request and ordinations and PERMANOVAs were performed using the *adonis* function in the *vegan* package.

## Results

### Free lists and salience indices

The 91 interviewees cited 197 ethnospecies or ethnogenera of which 33 were considered salient to the community (*p* < 0.05) (Table 2).

**Table 2:** List of the 33 most salient ethnospecies and ethnogenera of fish for Siribinha, Brazil

Ethnospecies	Probable academic scientific species	Salience	*p* value
Tainha	*Mugil curema*	0.717	< 0.001
Carapeba	*Eugerres brasilianus*	0.584	< 0.001
Robalão	*Centropomus undecimalis*	0.464	< 0.001
Robalo branco	*Centropomus parallelus*	0.386	< 0.001
Pescada branca	*Cynoscion leiarchus*	0.339	< 0.001
Pescada amarela	*Cynoscion acoupa*	0.330	< 0.001
Robalo espalmado	*Centropomus parallelus*	0.303	< 0.001
Curimã	*Mugil liza*	0.299	< 0.001
Vermelho	*Lutjanus purpureus*	0.294	< 0.001
Robalo*	*Centropomus* spp.	0.224	< 0.001
Corvina	*Micropogonias furnieri*	0.220	< 0.001
Bagre fidalgo	*Bagre bagre*	0.215	< 0.001
Bagre amarelo	*Sciades herzbergii*	0.206	< 0.001
Bagre griaçu	*Sciades proops*	0.191	< 0.001
Bagre do mangue	*Genidens barbus*	0.178	< 0.001
Xaréu	*Caranx hippos*	0.171	< 0.001
Cavala	*Scomberomorus cavalla*	0.165	< 0.001
Sardinha	*Opisthonema oglinum*	0.165	< 0.001
Pescada barracuda	*Sphyraena guachancho*	0.142	< 0.001
Bagre uruçu	*Apistor luniscutis*	0.136	< 0.001
Cação martelo	*Sphyrna* spp.	0.132	< 0.001
Sororoca	*Scomberomorus brasiliensis*	0.115	0.001
Capadinho	Unidentified	0.109	0.002
Cação*	Several species	0.107	0.003
Catana	*Trichiurus lepturus*	0.103	0.005
Bagre cagão	Unidentified	0.099	0.008
Badejo	*Mycteroperca bonaci*	0.088	0.025
Pescada*	*Cynoscion spp.*	0.088	0.026
Caçonete*	Several species	0.086	0.030
Arraia*	Several species	0.086	0.033
Roncador	*Ballistes vetulla*	0.084	0.039
Guaricema	*Caranx crysos*	0.083	0.041
Mirucaia	*Ctenosciaena gracilicirrhus*	0.083	0.042

The probable overlapping academic scientific species to the ethnospecies are indicated (identified by José Amorim dos Reis Filho/see main text for explanation). *Ethnogenera that encompasses many species. (See Additional file 1: Table S2 for complete list)

Of the 33 ethnospecies and ethnogenera, 28 overlapped with an academic scientific species or genus. The remaining five were left unidentified, either because the ethospecies/ethnogenus comprised many species from different genera or simply because the fisheries specialist could not ascertain the relationship between ethnospecies and academic scientific species.

The calculated salience indices for the recorded fish ranged from 0.717 (*tainha* – *Mugil curema*) to 0.0002 (*tainha meio olho* – *Mugil *sp*.*), with the former being cited in 85 out of 91 lists. Of the 197 fish ethnospecies/ethnogenera, 118 fish (60%) had low but statistically significant salience indices (0.022–0.0002). Because of the low salience indices, they cannot be considered salient ethnospecies/ethnogenera. A further 46 fish (23%) had insignificant *p*-values.

### Knowledge composition, gender and type of activity

**Fig. 1:**
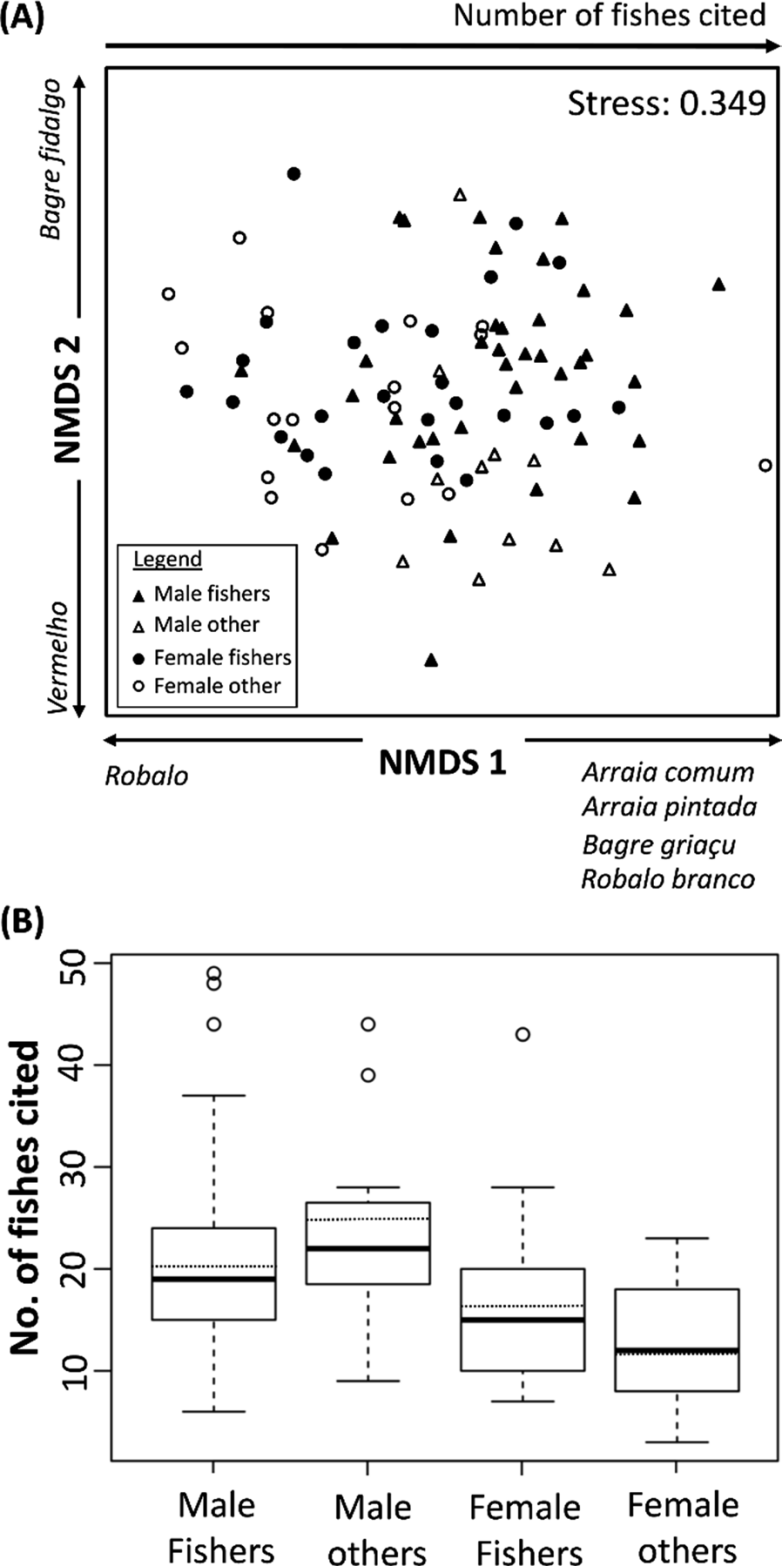
**A** Non-metric multidimensional ordination of ethnospecies and ethnogenera of fish cited during the free listing task by 91 interviewees from the fishing community of Siribinha, northeast Brazil. The arrows along the axes indicate significant positive or negative Pearson correlations (*p* > 0.00047 after Bonferroni correction for multiple comparisons) between individual fish or factors and the axes. For conciseness, only fish with r > 0.45 are shown. **B** Boxplots showing the median number of fish cited during the free listing tasks by gender and type of activity. Boxes enclose the median (thick line), the mean (thin dashed line), and the 25 and 75 percentiles

Our non-metric multivariate analysis ordination of the free list data shows very little structure in knowledge composition (Fig. 1A). However, the number of fish cited correlated positively with NMDS axis 1. Also, seven ethnospecies exhibited strong correlations (r > 0.45, *p* < 0.00047) with NMDS axes 1 or 2 (Fig. 1A) and are among the list of the 33 most salient fish ethnospecies to the community (Table 2). Additionally, a PERMANOVA showed that gender was a significant factor influencing the number of fish cited (*F*_1,90_ = 3.39, *p* = 0.005), but type of activity (*F*_1,90_ = 1.255, *p* = 0.184) and the interactions (*F*_1,90_ = 1.282, *p* = 0.110) between gender and type of activity were not significant.

In terms of the number of fish cited, male non-fisher interviewees cited the highest mean number of fish, followed by male fishers, female fishers, and female non-fishers (Fig. 1B), although intergroup comparisons were not statistically significant.

### Triad tasks

**Fig. 2:**
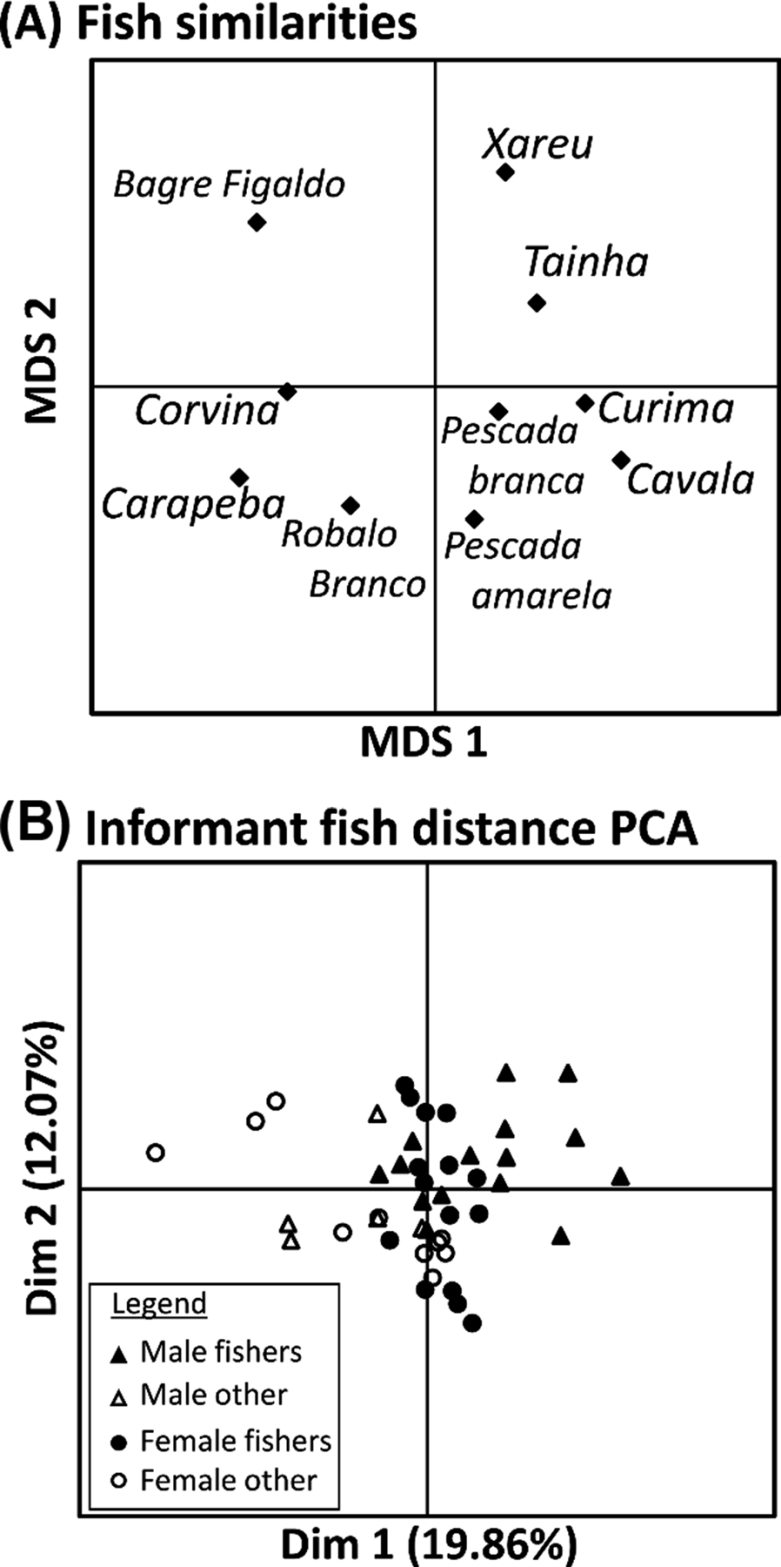
**A** Non-metric multidimensional scaling (MDS) ordinations of fish similarity judgments as determined by triad tasks from 45 interviewees from an artisanal fishing community in Siribinha, northeast Brazil. **B** The inter-interviewee fish distance matrix correlations were analyzed with Principal Components Analyses to check for consensus within and across groups of interviewees (male fishers, male non-fishers, female fishers, and female non-fishers)

Our non-metric multidimensional scaling ordinations (Fig. 2) based on the interviewees’ triad task results showed no distinct groupings in the 10 fish (Fig. 2A). Bagre fidalgo and xaréu stood out from the others, mainly for their morphological traits, behaviour or taste, used by the interviewees to distinguish them from the other ethnospecies. Statements like the following were common:

L.42: Xaréu and bagre are the most different ones/ they are not so tasty.

E.33: I don’t like xaréu so much/ it has worms/ we use it for bait mainly/(…) it’s a scaleless fish/(…) it smells bad/ there’s no taste/(…) bagre fidalgo has no scales/just leather.

E.68: Xaréu *and bagre are two ****carregado**** fish (commonly used term in many artisanal fishing communities in Brazil to discriminate fish that should be avoided for consumption by people with wounds, measles, tumors, skin rash, and other maladies, or by women after childbirth or undergoing menstruation, because they are believed to cause inflammation *[44])*.*

D.49: *Almost no one likes xaréu/ its meat is dark/ it has a third quality meat/ (…) bagre is different from the others/ it's out of the ordinary.*

PCA results on the inter-interviewee correlation matrix were used to determine if there was a single underlying model for fish categorization (Fig. 2B) and returned a first factor to second factor eigenvalue ratio of 1.563, while the variation explained by the first two axes were 19.86% and 12.07% respectively. There were also negative loadings on the first factor (see Additional file 1: Table S3). These results indicate weak agreement among interviewees, and suggest a lack of a cultural consensus model.

Our PERMANOVA analyses on the PCA scores of the inter-interviewee correlation matrices showed that gender and type of activity were both significant factors for fish ethnotaxonomy (*p* = 0.005 and < 0.001 respectively) (Table 3). However, the interactions between these factors were not significant (*p* = 0.328) (Table 3).

**Table 3:** F-statistics and significance level (*p*) of PERMANOVA comparisons of inter-interviewee correlation matrices of fish distances obtained from triad

	*F*_1,41_	*p*
Gender	2.287	0.005
Occupation	4.419	< 0.001
Gender & Occupation	1.080	0.328

When we analyzed the proportion of triads in which each participant agreed with the “modal response”, there was no clear and significant distinction between the agreement of the 45 participants in the categorization of fish (one-way ANOVA: F = 0.391; *p* = 0.679).

## Discussion

### Salience indices

The salient ethnospecies or ethnogenera considered by the 91 participants in the free listing task (*p* < 0.001–0.042) are also, according to them, the most fished ones in the community. Therefore, the community members naturally have more contact with them.

The 118 fish ethnospecies with lower salience values (0.022–0.0002) but significant *p* values can be considered idiosyncrasies [e.g. 36]. Such items were cited at the end of the free lists or were known by very few people, or yet very few people knew these fish by this name (e.g., when a fish has many different common names and some of them are not widely spread around). For these reasons, items with low saliency have been interpreted in the literature as being of little or no cultural importance, or even mistakes [36]. Items that were cited only once (73 fish out of 197–37%) are also considered idiosyncratic.

We tried to decrease the limitations and biases in the method as much as possible, but some methodological decisions have influenced the results in a relevant manner. For instance, when we asked for scrutinization on the ethnogenus, these were excluded when the interviewees mentioned ethnospecies linked to them. Therefore, it is possible that, by doing so, we deflated the salience indices of some ethnogenus, and inflated those of some ethnospecies.

### The role of gender and type of activity in ethnotaxonomy

Gender roles are very important in artisanal fishing communities in Brazil [e.g., 39–40], and the Siribinha community is no exception. Reported differences in intracultural knowledge about the environment in several studies suggest a link between gender and type of activity or social role [41, 42]. Our data support this association, as the average number of fish cited during the free listing was higher among men than among women. Since fishing is performed primarily by men while women play a predominant role in collecting shellfish, it is not surprising that men have a greater knowledge about fish (as reflected in a greater average number of citations).

Follow-up studies could perhaps examine if this could be related to direct contact with fish (and if so, if women would have a greater knowledge of shellfish species than men). Tng et al. [21], for example, performed an ethnobotanical study in Siribinha and found that female and male traditional experts possess a different set of plant use knowledge, with women generally citing more food and medicinal plants, and men citing more wood and fiber plants. Therefore, intracultural variation in plant knowledge and probably fish knowledge in Siribinha is also related to social role and type of activity.

Nevertheless, it is still not clear why we did not find male-fishers citing significantly more fish than non-fishers. Even though we did not find statistically significant intergroup comparisons, this is still a relevant and intriguing result, since in this specific case, type of subsistence activity does not clearly relate to what might be expected based on the knowledge of local people, as assessed through the number of fish cited in a free list. It is fair to say, however, that this is a predominantly fishing village, and even though many inhabitants do not depend on fishing directly for their livelihoods, their lives are closely intertwined with the fishing culture. The vast majority engage in fishing in their free time (either for self-consumption or for leisure) and/or live in the same household or have a close relationship (married to/mother or father/son or daughter etc.) with a fisherman or a fisherwoman.

We acknowledge that age plays a considerable role in the distribution of indigenous and local knowledge [22–24], alongside gender and type of activity, however we were not able to account for many of the interviewees’ age, hindering the age analysis on both free lists and triad tasks.

### Lack of cultural consensus

Debates about indigenous and local knowledge often treat knowledge of a community as homogenous [15]. This tendency can be further reinforced by ethnotaxonomic traditions of emphasizing cross-cultural stability and universality of categories [5]. But we encountered a very different pattern, since a lack of cultural consensus was observed. That is, even within relatively homogeneous cultural groups, there was great variation of response, agreeing with findings of Boster and Johnson [25].

In the MDS analyses of both the free list and triad task results, we did not find cultural consensus regarding the similarity of fish ethnospecies and their categorization. This may be in part due to the different criteria used for distinguishing fish. Most of them distinguished fish by phenotype, but some of them also used other criteria, such as flavor, difficulties to catch, lack of familiarity, specific locations for catching and value or marketability. The lack of cultural consensus might be also a result of the production of individual knowledge (innovations), recent information inputs (immigration), changes of the original information (mutations) or low mnemonic relevance [36].

Nevertheless, the triad task shows a limitation. The number of triads used in a task grows exponentially, the higher the amount of items used. In a Lambda 2 design, 10 items generate 30 triads. In the same design, 25 items generate 200 triads [35], which would make an interview last a few hours. Even if we had opted to use a Lambda 1 design, performing an interview with the 33 most salient ethnospecies and ethnogenera, would make the interview too long, affecting the quality of the interviewees’ responses. If that would have been possible, nevertheless, we would have probably observed a few clusters in the ordinations of fish (Fig. 2A), for instance, a cluster with some of the six ethnospecies of *bagre* (catfish) that are part of the most salient ethnospecies and ethnogenera, or another one with the three ethnospecies of *robalo* (snook) that are part of that list. However, we believe that would not be enough for the structure of the agreement to be explained by a single factor solution, and, thus, a lack of cultural consensus would still be found.

Additionally, we did not find a common conceptual organization of the ethnospecies and ethnogenera. These results varied substantially from the findings of Ross et al. [15], who studied plant categorization by the Tzotzil Maya from Zinacantán (Highlands of Chiapas, Mexico), and Medin and colleagues [7], who investigated the categorization of freshwater fish by Native American and majority-culture fish experts from north central Wisconsin (USA). Nevertheless, our results reinforce the argument of Ross and colleagues [15] for caution about beginning ethnobiological research with the assumption that a community possesses a single cultural consensus model, as is sometimes done.

In a similar vein, Vandebroek [43] argues that it is challenging to extrapolate knowledge of individual participants to a community or cultural group. Therefore, the careful selection of participants deserves considerable attention before the start of fieldwork. For instance, interviewing only the oldest or most experienced traditional experts in a community does not imply that representative (general and commonly-shared) cultural information will be recorded. On the contrary, when the development of expertise results in learning many alternate devices or bases for structuring a domain, the experts will be more variable in their responses than novices and so will tend to deviate more often from a consensus [25].

## Conclusions

Indigenous and local communities are strongholds of ethnobiological knowledge but establishing if there is cultural consensus related to this knowledge within them has been a challenge in many ethnobiological studies [e.g. [7, 15, 17]. Our ethnobiological study in Siribinha, an artisanal fishing community in northeast Brazil, revealed that the community has a rich knowledge of fish which is patterned by gender. We also found that there was no cultural consensus in the ways fish are classified by the community members. These observations call for caution in making assumptions that a given local community would have a single cultural consensus model in classifying the organisms that they encounter in their daily lives.

The lack of "cultural consensus" does not mean, however, that there are no patterns in the community’s knowledge or even that the responses are only random noise. We therefore need to address ethnobiological knowledge in ways that are sensitive to issues of social stratification (e.g., along gender and type of activity) and epistemic diversity within communities without assuming that there is nothing to be said about communities more generally. In this sense, ethnobiologists are challenged to avoid treating communities either as monolithic epistemic units or as entirely fragmented collections of individuals.

## Supplementary Information

**Additional file 1:** Supplementary Material.

## Data Availability

The datasets used and/or analyzed during the current study are available from the corresponding author on reasonable request, provided that confidentiality and other ethical issues are respected and maintained.

## Abbreviations

CCM: Cultural consensus model
NMDS: Non-metric multidimensional scaling
PCA: Principal components analysis
PERMANOVA: Permutated multivariate analysis of variance
