# Supplementary material for: Cultural consensus and intracultural diversity in ethnotaxonomy: lessons from a fishing community in Northeast Brazil
Source: J Ethnobiol Ethnomed. 2022 Mar 27;18:25. doi: 10.1186/s13002-022-00522-y (PMC8962115; doi:10.1186/s13002-022-00522-y)
Supplement: Supplementary file 1 — Additional file 1: Supplementary Material. [file 13002_2022_522_MOESM1_ESM.docx]

Supplementary Material

1. Portuguese originals from the translated excerpts

L.42: *xareu e bagre são os mais diferentes/ são ruins de comer.*

*Xareu and bagre are the most different ones/ they are not so tasty.*

E.33: *não gosto muito do xareu/ tem verme/ a gente usa mais pra isca/ (...) é peixe de couro/ (...) tem cheiro ruim/ não tem gosto/ (...) bagre fidalgo não tem escama/ só couro.*

*I don’t like xareu so much/ it has worms/ we use it for bait mainly/ (…) it’s a scaleless fish/ (…) it smells bad/ there’s no taste/ (…) bagre fidalgo has no scale/ just leather.*

E.68: *xareu e bagre são dois peixes carregados.*

*Xareu and bagre are two carregado fish.*

D.49: *o xareu quase ninguém gosta/ a carne é escura/ de terceira qualidade/ (...) bagre é diferente dos outros/ é fora do normal.*

*Almost no one likes xareu/ its meat is dark/ of third quality/ (…) bagre is different from the others/ it's out of the ordinary.*

Table S1. Triad Task Photographs

| **Ethnospecies (scientific name)** | **Photographs** |
| --- | --- |
| Bagre Fidalgo (*Bagre bagre*) | 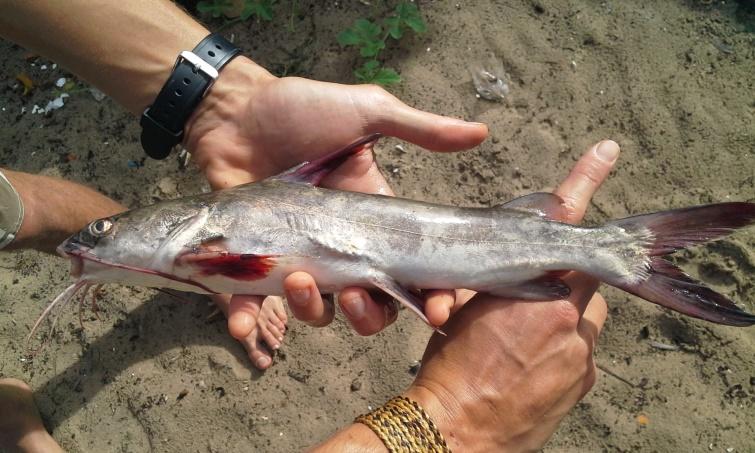  Photo by author |
| Carapeba (*Eugerres brasilianus*) | 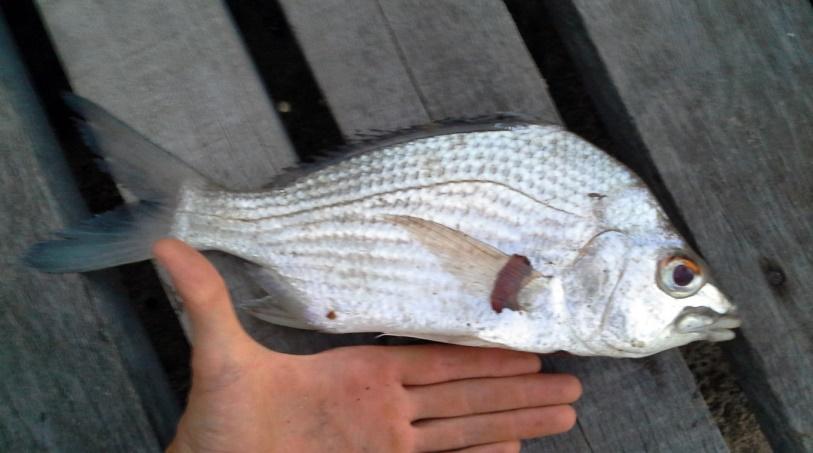  Photo by author |
| Cavala (*Scomberomorus cavala*) | *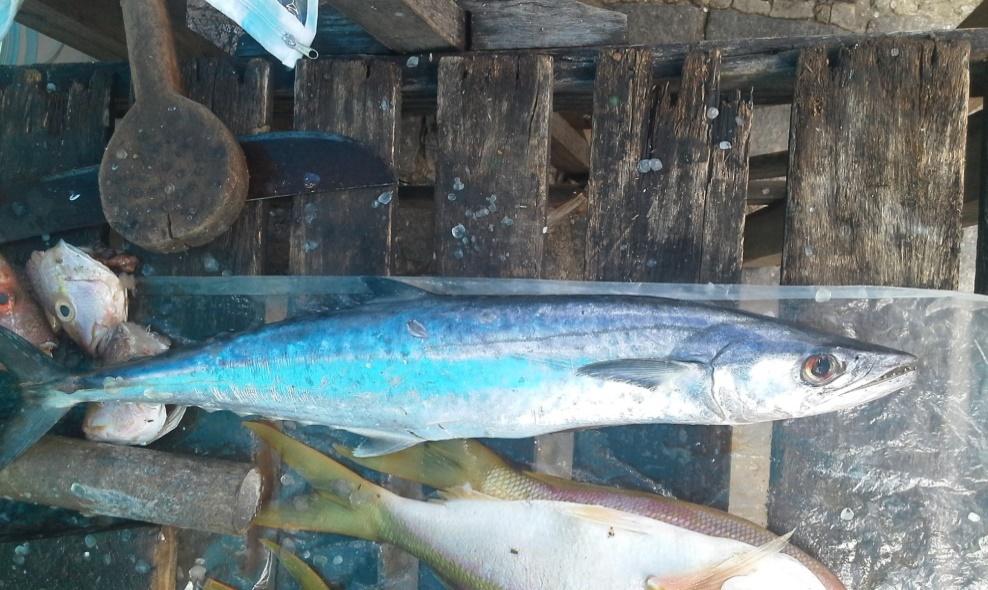*  Photo by author |
| Corvina (*Micropogonias furnieri*) | *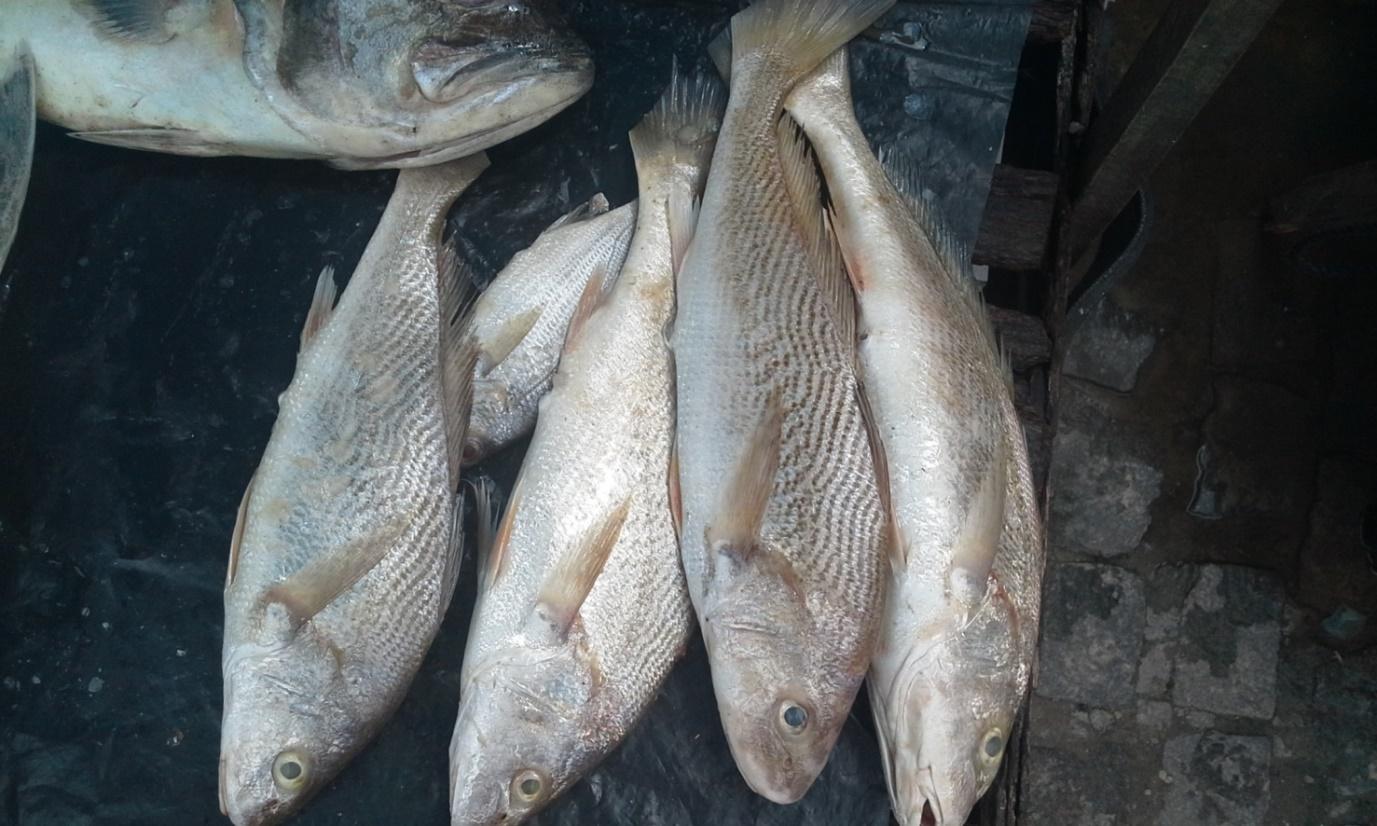*  Photo by author |
| Curimã  (*Mugil liza*) | *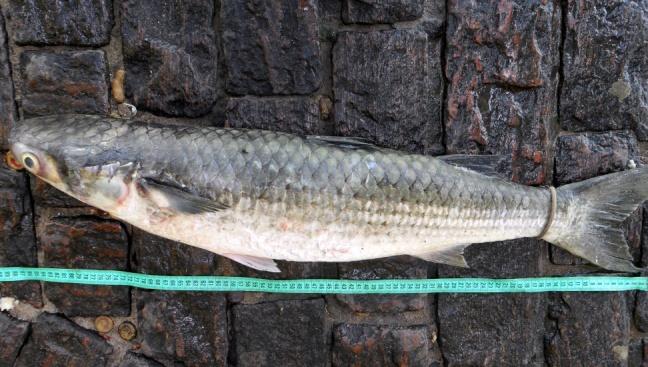*  Photo by José Amorim dos Reis Filho |
| Pescada amarela (*Cynoscion acoupa*) | *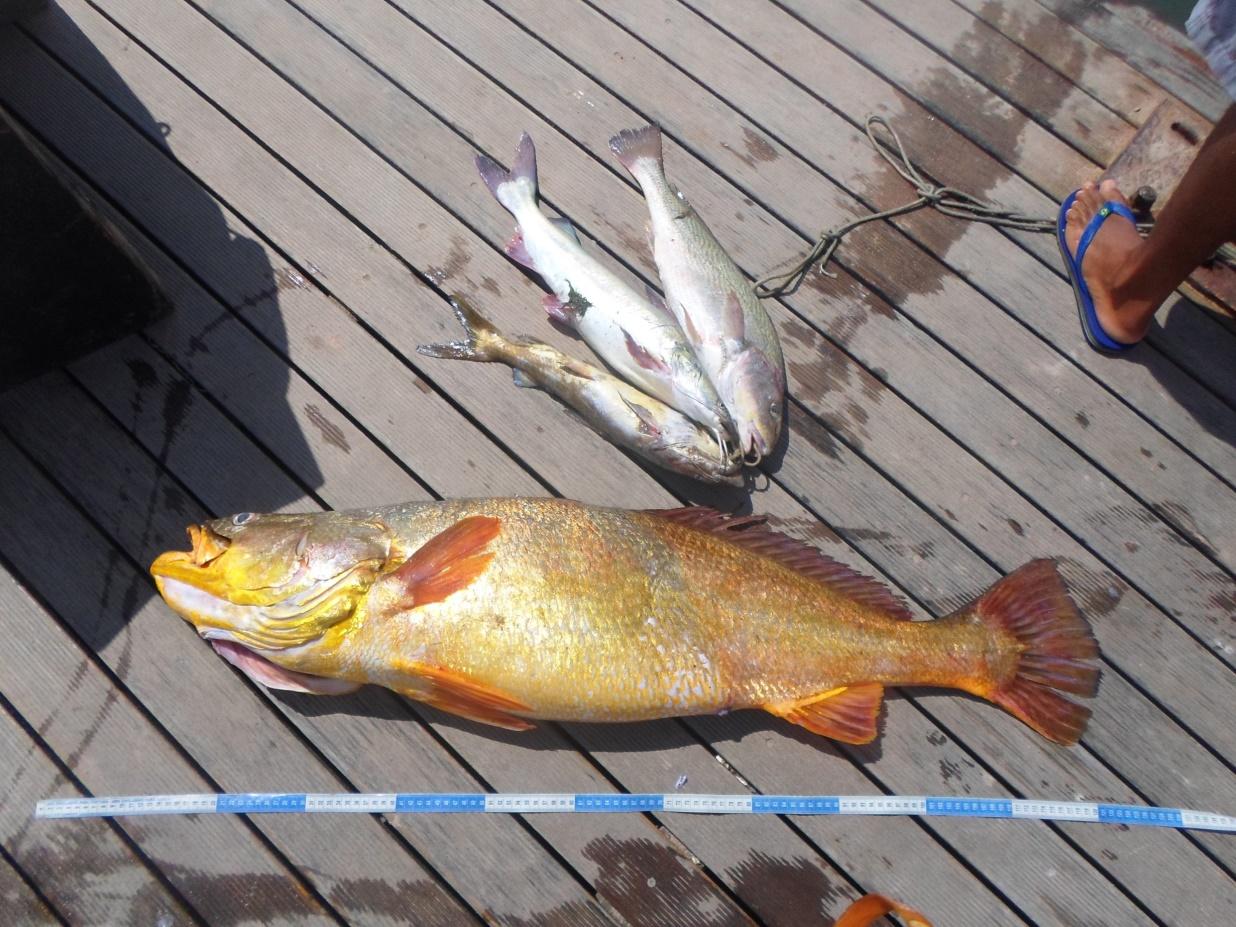*  Photo by José Amorim dos Reis Filho |
| Pescada branca (*Cynoscion leiarchus*) | 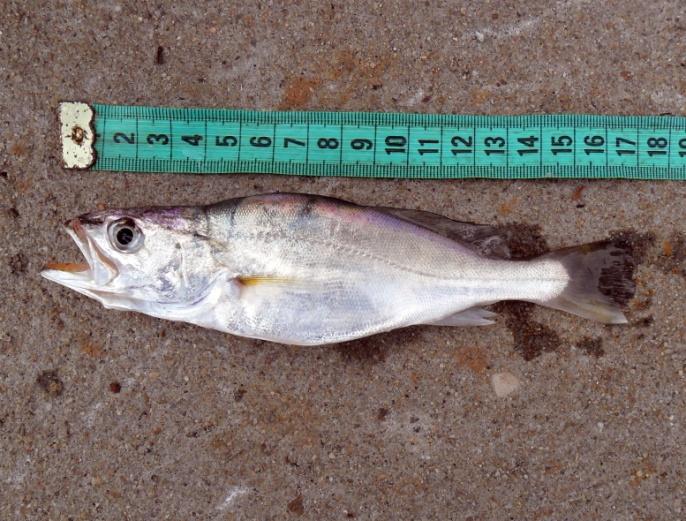  Photo by José Amorim dos Reis Filho |
| Robalo branco (*Centropomus parallelus*) | *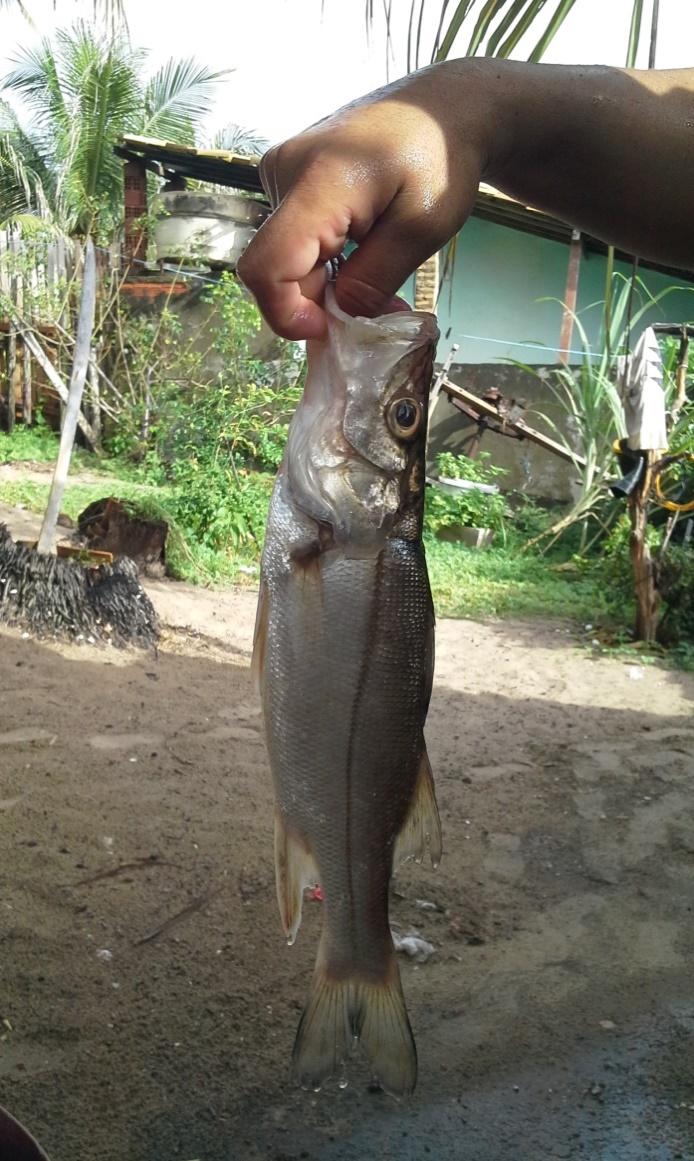*  Photo by author |
| Tainha  (*Mugil curema*) | *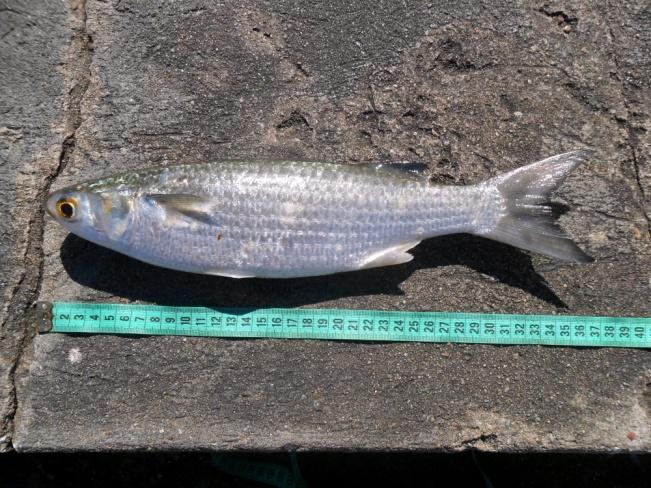*  Photo by José Amorim dos Reis Filho |
| Xareu  (*Caranx hippos*) | *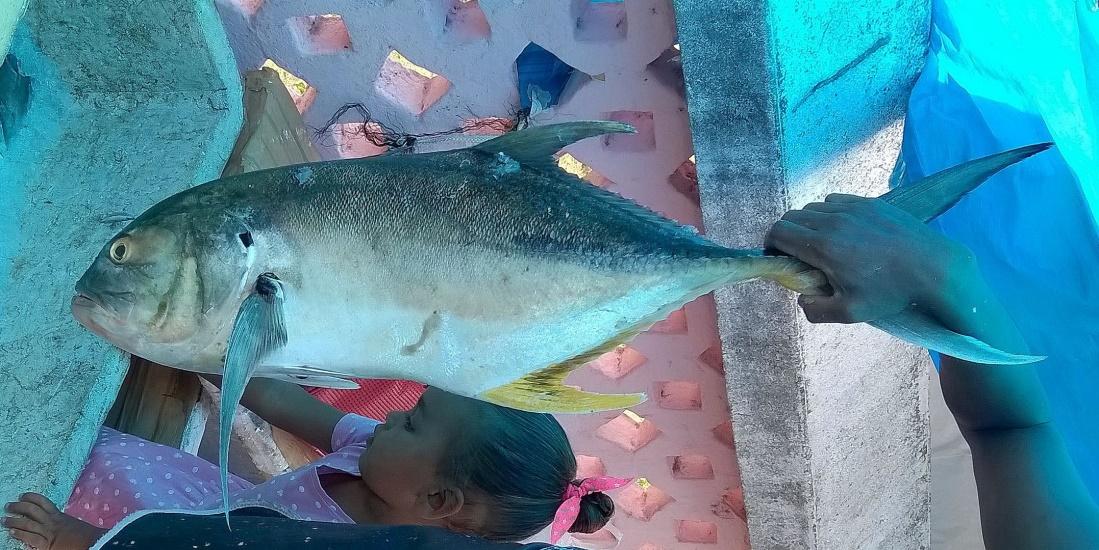*  Photo by author |


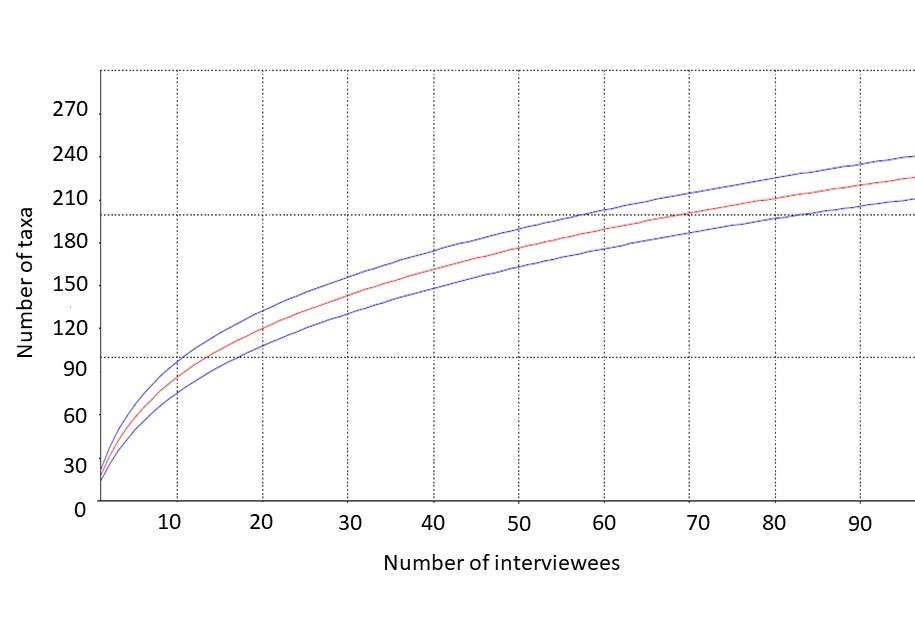


Fig. S1. Free List Rarefied Ethnospecies-interviewee Curve Analysis

Table S2. Complete list of fish saliency according to the participants of the free listing task in Siribinha, Brazil

|  | Ethnospecies | Probable academic scientific species | Salience | *p-*value |
| --- | --- | --- | --- | --- |
| 1 | Tainha | *Mugil curema* | 0.717 | <0.001 |
| 2 | Carapeba | *Eugerres brasilianus* | 0.584 | <0.001 |
| 3 | Robalão | *Centropomus undecimalis* | 0.464 | <0.001 |
| 4 | Robalo branco | *Centropomus parallelus* | 0.386 | <0.001 |
| 5 | Pescada branca | *Cynoscion leiarchus* | 0.339 | <0.001 |
| 6 | Pescada amarela | *Cynoscion acoupa* | 0.330 | <0.001 |
| 7 | Robalo espalmado | *Centropomus parallelus* | 0.303 | <0.001 |
| 8 | Curimã | *Mugil liza* | 0.299 | <0.001 |
| 9 | Vermelho | *Lutjanus purpureus* | 0.294 | <0.001 |
| 10 | Robalo | *Centropomus spp.* | 0.224 | <0.001 |
| 11 | Corvina | *Micropogonias furnieri* | 0.220 | <0.001 |
| 12 | Bagre fidalgo | *Bagre bagre* | 0.215 | <0.001 |
| 13 | Bagre amarelo | *Sciades herzbergii* | 0.206 | <0.001 |
| 14 | Bagre griaçu | *Sciades proops* | 0.191 | <0.001 |
| 15 | Bagre do mangue | *Genidens barbus* | 0.178 | <0.001 |
| 16 | Xareu | *Caranx hippos* | 0.171 | <0.001 |
| 17 | Cavala | *Scomberomorus cavalla* | 0.165 | <0.001 |
| 18 | Sardinha | *Opisthonema oglinum* | 0.165 | <0.001 |
| 19 | Pescada barracuda | *Sphyraena guachancho* | 0.142 | <0.001 |
| 20 | Bagre uruçu | *Apistor luniscutis* | 0.136 | <0.001 |
| 21 | Cação martelo | *Sphyrna sp.* | 0.132 | <0.001 |
| 22 | Sororoca | *Scomberomorus brasiliensis* | 0.115 | 0.001 |
| 23 | Capadinho | Unidentified | 0.109 | 0.002 |
| 24 | Cação | Several species | 0.107 | 0.003 |
| 25 | Catana | *Trichiurus lepturus* | 0.103 | 0.005 |
| 26 | Bagre cagão | Unidentified | 0.099 | 0.008 |
| 27 | Badejo | *Mycteroperca bonaci* | 0.088 | 0.025 |
| 28 | Pescada | *Cynoscion spp.* | 0.088 | 0.026 |
| 29 | Caçonete | Several species | 0.086 | 0.030 |
| 30 | Arraia | Several species | 0.086 | 0.033 |
| 31 | Roncador | *Ballistes vetulla* | 0.084 | 0.039 |
| 32 | Guaricema | *Caranx crysos* | 0.083 | 0.041 |
| 33 | Mirucaia | *Ctenosciaena gracilicirrhus* | 0.083 | 0.042 |
| 34 | Cação lixa | *Ginglymostoma cirratum* | 0.080 | 0.056 |
| 35 | Caranha | *Lutjanus cyanopterus* | 0.077 | 0.073 |
| 36 | Mero | *Epinephelus itajara* | 0.075 | 0.089 |
| 37 | Pescada cumbucu | *Cynoscion sp.* | 0.072 | 0.121 |
| 38 | Bagre | Several species | 0.068 | 0.158 |
| 39 | Barbudo | *Polydactilus virginicus* | 0.067 | 0.167 |
| 40 | Cação gaia preta | *Carcharhinus sp.* | 0.064 | 0.203 |
| 41 | Cação rabo seco | *Rhizoprionodon sp.* | 0.063 | 0.229 |
| 42 | Arraia pintada | *Aetobatus narinari* | 0.060 | 0.276 |
| 43 | Arraia morcego | *Rhinoptera bonasus* | 0.056 | 0.343 |
| 44 | Robalo cachorro | *Centropomus sp.* | 0.055 | 0.363 |
| 45 | Pescadinha | *Stellifer sp.* | 0.055 | 0.367 |
| 46 | Papa terra | *Stellifer sp.* | 0.049 | 0.505 |
| 47 | Cação viola | *Pseudobatos percellens* | 0.046 | 0.433 |
| 48 | Dentão | *Lutjanus sp.* | 0.043 | 0.366 |
| 49 | Cutupá | Unidentified | 0.042 | 0.342 |
| 50 | Olho de boi | *Seriola dumerili* | 0.041 | 0.323 |
| 51 | Bonito | *Euthinus alleteratus* | 0.040 | 0.302 |
| 52 | Beiju pirá | *Rachycentron canadum* | 0.039 | 0.286 |
| 53 | Bagre branco | *Sciades sp.* | 0.038 | 0.278 |
| 54 | Traíra | *Hoplias malabaricus* | 0.038 | 0.276 |
| 55 | Boca larga | Unidentified | 0.038 | 0.268 |
| 56 | Robalo corcunda | *Pomadasys crocro* | 0.038 | 0.260 |
| 57 | Pampo | *Trachinotus sp.* | 0.037 | 0.245 |
| 58 | Azeiteira | *Mugil sp.* | 0.037 | 0.243 |
| 59 | Arraia jamanta | *Mobula sp.* | 0.036 | 0.232 |
| 60 | Baiacu | *Sphoeroides sp.* | 0.035 | 0.217 |
| 61 | Robalinho | *Centropomus* | 0.035 | 0.213 |
| 62 | Vermelha | *Lutjanus alexandrei* | 0.034 | 0.201 |
| 63 | Amoreia | *Dormitator maculatus* | 0.032 | 0.169 |
| 64 | Carapicum | *Eucinostomus sp.* | 0.032 | 0.155 |
| 65 | Galo | *Selene vômer* | 0.031 | 0.149 |
| 66 | Graçaim | *Caranx lugubris* | 0.031 | 0.148 |
| 67 | Xira | *Haemulon plumieri* | 0.031 | 0.145 |
| 68 | Tilápia | *Oreochromis niloticus* | 0.031 | 0.144 |
| 69 | Caramuru | *Gymnothorax sp.* | 0.031 | 0.142 |
| 70 | Dourado | *Coryphaena hippurus* | 0.030 | 0.136 |
| 71 | Piranha | *Serrasalmus brandtii* | 0.030 | 0.136 |
| 72 | Gaiuba | *Ocyurus chrysurus* | 0.030 | 0.129 |
| 73 | Cangurupim | *Megalops atlanticus* | 0.028 | 0.102 |
| 74 | Atum | *Thunnus spp.* | 0.027 | 0.092 |
| 75 | Cação galinha | Unidentified | 0.026 | 0.089 |
| 76 | Arraia comum | *Dasyatis spp.* | 0.026 | 0.081 |
| 77 | Cação tigre | *Galeocerdo cuvier* | 0.025 | 0.069 |
| 78 | Carapebinha | *Diapterus sp.* | 0.024 | 0.064 |
| 79 | Cação branco | *Rizoprionodon sp.* | 0.022 | 0.052 |
| 80 | Paru | *Pomacanthus sp.* | 0.022 | 0.048 |
| 81 | Suia | *Symphurus sp.* | 0.021 | 0.044 |
| 82 | Tinga | *Diapterus sp.* | 0.020 | 0.034 |
| 83 | Cação bico doce | *Rhizoprionodon sp.* | 0.018 | 0.026 |
| 84 | Peixe boi | *Trichechus manatus* | 0.018 | 0.026 |
| 85 | Tubarão | Several species | 0.018 | 0.024 |
| 86 | Arraia mijona | *Dasyatis sp.* | 0.018 | 0.023 |
| 87 | Bagre upemba | Unidentified | 0.017 | 0.021 |
| 88 | Corró | *Geophagus brasiliensis* | 0.017 | 0.020 |
| 89 | Vermelho dentão | *Lutjanus sp.* | 0.017 | 0.019 |
| 90 | Arraia gereba | Unidentified | 0.016 | 0.018 |
| 91 | Pescada jambuiu | *Cynoscion sp.* | 0.016 | 0.016 |
| 92 | Vermelho rabo aberto | *Ocyurus chrysurus* | 0.016 | 0.016 |
| 93 | Aracanguira | *Selene setapinnis* | 0.016 | 0.016 |
| 94 | Bagre do rio | Unidentified | 0.015 | 0.014 |
| 95 | Cioba | *Lutjanus analis* | 0.015 | 0.013 |
| 96 | Arraia branca | *Dasyatis sp.* | 0.014 | 0.012 |
| 97 | Solteira | *Oligoplites sp.* | 0.014 | 0.012 |
| 98 | Cação cabeça lisa | Unidentified | 0.013 | 0.009 |
| 99 | Bagre jundiá | *Rhamdia quelen* | 0.010 | 0.005 |
| 100 | Baleia | *Megaptera novaeangliae* | 0.010 | 0.005 |
| 101 | Garapau | *Chloroscombrus chrysurus* | 0.010 | 0.004 |
| 102 | Cascudinha | Unidentified | 0.010 | 0.004 |
| 103 | Niquim | *Thalassophryne sp.* | 0.010 | 0.004 |
| 104 | Robalo coco | *Pomadasys corvinaeformis* | 0.010 | 0.004 |
| 105 | Bagre da praia | Unidentified | 0.009 | 0.004 |
| 106 | Bagre do mar | Unidentified | 0.009 | 0.004 |
| 107 | Tainha olho de fogo | *Mugil sp.* | 0.009 | 0.004 |
| 108 | Trambitara | Unidentified | 0.009 | 0.004 |
| 109 | Bagre preto | Unidentified | 0.009 | 0.004 |
| 110 | Cação da areia | *Pseudobatos percellens* | 0.009 | 0.004 |
| 111 | Peixe porco | *Balistes vetula* | 0.009 | 0.004 |
| 112 | Robalo espada | *Centropomus undecimalis* | 0.009 | 0.004 |
| 113 | Bagre mandí | Unidentified | 0.009 | 0.004 |
| 114 | Enxova | *Pomatomus saltatrix* | 0.009 | 0.003 |
| 115 | Barana | *Albula vulpes* | 0.009 | 0.003 |
| 116 | Pescada dentão | *Cynoscion microlepidotus* | 0.009 | 0.003 |
| 117 | Bagre barbudo | *Bagre bagre* | 0.008 | 0.003 |
| 118 | Bagre cabeçudo | Unidentified | 0.008 | 0.003 |
| 119 | Dorminhoco | *Lobotes surinamensis* | 0.008 | 0.003 |
| 120 | Pescada olho de conta | *Cynoscion sp.* | 0.008 | 0.003 |
| 121 | Garoupa | *Epinephelus sp.* | 0.008 | 0.003 |
| 122 | Bagre cangatá | Unidentified | 0.008 | 0.002 |
| 123 | Ariocó | *Lutjanus synagris* | 0.008 | 0.002 |
| 124 | Avacora | *Thunnus sp.* | 0.008 | 0.002 |
| 125 | Pescada guete | *Cynoscion sp.* | 0.008 | 0.002 |
| 126 | Robalo falcão | *Centropomus sp.* | 0.008 | 0.002 |
| 127 | Bagre veleiro | *Bagre bagre* | 0.008 | 0.002 |
| 128 | Giruna | Unidentified | 0.007 | 0.002 |
| 129 | Língua de vaca | *Cynoglossidae* | 0.007 | 0.002 |
| 130 | Cação espada | Unidentified | 0.007 | 0.002 |
| 131 | Cação mouriço | *Mustelus sp.* | 0.007 | 0.002 |
| 132 | Linguado | *Achiridae sp.* | 0.007 | 0.002 |
| 133 | Gaibira | *Oligoplites sp.* | 0.007 | 0.002 |
| 134 | Piau | *Leporinus sp.* | 0.007 | 0.002 |
| 135 | Arraia amarela | *Dasyatis sp.* | 0.007 | 0.002 |
| 136 | Tucunaré | *Cichla sp.* | 0.007 | 0.002 |
| 137 | Mututuca | *Mirychthys sp.* | 0.006 | 0.002 |
| 138 | Voador | *Parexocoetus sp.* | 0.006 | 0.001 |
| 139 | Bagre azul | Unidentified | 0.006 | 0.001 |
| 140 | Arraia manteiga | *Gymnura micrura* | 0.006 | 0.001 |
| 141 | Vermelho boca negra | Unidentified | 0.006 | 0.001 |
| 142 | Cocelo | Unidentified | 0.006 | 0.001 |
| 143 | Pescada perna de moça | *Cynoscion sp.* | 0.006 | 0.001 |
| 144 | Pescada de água doce | *Plagioscion squamosissimus* | 0.005 | 0.001 |
| 145 | Pescada vermelha | *Cynoscion breviceps* | 0.005 | 0.001 |
| 146 | Tapa | *Citharichthys sp.* | 0.005 | 0.001 |
| 147 | Budião azul | *Scarus trispinosus* | 0.005 | 0.001 |
| 148 | Gutupá | Unidentified | 0.005 | 0.001 |
| 149 | Cação treme treme | *Narcine brasiliensis* | 0.005 | 0.001 |
| 150 | Mututuca pintada | *Mirychthys sp.* | 0.005 | 0.001 |
| 151 | Pocomon | *Amphichthys cryptocentrus* | 0.005 | 0.001 |
| 152 | Cação panã | *Sphyrna sp.* | 0.005 | 0.001 |
| 153 | Caboge | *Hoplosternum littorale* | 0.005 | 0.001 |
| 154 | Cara suja | Unidentified | 0.004 | 0.001 |
| 155 | Pescada de água salgada | *Cynoscion sp.* | 0.004 | 0.001 |
| 156 | Vermelho amarelo | *Ocyurus chrysurus* | 0.004 | 0.001 |
| 157 | Curimatá | *Prochilodus sp.* | 0.004 | <0.001 |
| 158 | Baiacu xareu | *Colomesus sp.* | 0.004 | <0.001 |
| 159 | Paramirim | *Rhomboplites aurorubens* | 0.004 | <0.001 |
| 160 | Marlim | *Xyphia sp.* | 0.004 | <0.001 |
| 161 | Olho de cão | *Priacanthus arenatus* | 0.004 | <0.001 |
| 162 | Vermelho branco | *Lutjanus sp.* | 0.004 | <0.001 |
| 163 | Peixe morcego | *Ogcocephalus sp.* | 0.003 | <0.001 |
| 164 | Jabú | *Cephalopholis fulva* | 0.003 | <0.001 |
| 165 | Cavalinha | *Scomberomorus sp.* | 0.003 | <0.001 |
| 166 | Golfinho | *Tursiops truncatus* | 0.003 | <0.001 |
| 167 | Cação amarelinho | Unidentified | 0.003 | <0.001 |
| 168 | Aratubaia | Unidentified | 0.002 | <0.001 |
| 169 | Cavala aipim | *Acanthocybium sp.* | 0.002 | <0.001 |
| 170 | Lauê | Unidentified | 0.002 | <0.001 |
| 171 | Iuiú | *Hoplerytrinus unitaeniatus* | 0.002 | <0.001 |
| 172 | Peixe agulha | *Hemiramphus sp.* | 0.002 | <0.001 |
| 173 | Cavala manteiga | Unidentified | 0.002 | <0.001 |
| 174 | Pacu | *Myleus sp.* | 0.002 | <0.001 |
| 175 | Vermelho saramonete | *Pseudupeneus maculatus* | 0.002 | <0.001 |
| 176 | Muçum | *Synbranchus marmoratus* | 0.002 | <0.001 |
| 177 | Paru branco | *Chaetodipterus faber* | 0.001 | <0.001 |
| 178 | Caranha do rio | Unidentified | 0.001 | <0.001 |
| 179 | Sauara | Unidentified | 0.001 | <0.001 |
| 180 | Carapebota | *Diapterus sp.* | 0.001 | <0.001 |
| 181 | Corongo | Unidentified | 0.001 | <0.001 |
| 182 | Mandí | *Pimelodus maculatus* | 0.001 | <0.001 |
| 183 | Pinima | *Sphoeroides sp.* | 0.001 | <0.001 |
| 184 | Tainha curiaçu | *Mugil sp.* | 0.001 | <0.001 |
| 185 | Caranha do mar | *Lutjanus cyanopterus* | 0.001 | <0.001 |
| 186 | Pirarucu | *Arapaima giga* | 0.001 | <0.001 |
| 187 | Tupa | Unidentified | 0.001 | <0.001 |
| 188 | Lambarí | *Astyanax sp.* | 0.001 | <0.001 |
| 189 | Acarí | *Astronotus ocellatus* | 0.001 | <0.001 |
| 190 | Arraia chapeu de couro | Unidentified | 0.001 | <0.001 |
| 191 | Cação de couro | Unidentified | 0.001 | <0.001 |
| 192 | Tainha patriaçu | *Mugil sp.* | 0.001 | <0.001 |
| 193 | Milongo | Unidentified | <0.001 | <0.001 |
| 194 | Suia da lama | *Symphurus sp.* | <0.001 | <0.001 |
| 195 | Pescada boca larga | Unidentified | <0.001 | <0.001 |
| 196 | Suia do rio | *Symphurus sp.* | <0.001 | <0.001 |
| 197 | Tainha meio olho | *Mugil sp.* | <0.001 | <0.001 |

Table S3. PCA loadings for the inter-interviewee fish distance matrix correlations (see main text Figure 2B).

| LOADINGS | PC 1 | PC 2 |
| --- | --- | --- |
| 1 | -0.111 | -0.101 |
| 2 | -0.023 | -0.018 |
| 3 | 0.011 | -0.440 |
| 4 | -0.058 | -0.157 |
| 5 | 0.038 | 0.058 |
| 6 | 0.220 | -0.272 |
| 7 | -0.206 | 0.147 |
| 8 | 0.176 | -0.140 |
| 9 | 0.306 | -0.181 |
| 10 | -0.183 | -0.010 |
| 11 | 0.036 | 0.177 |
| 12 | -0.291 | -0.095 |
| 13 | 0.135 | 0.091 |
| 14 | 0.157 | -0.014 |
| 15 | 0.219 | -0.068 |
| 16 | -0.361 | -0.012 |
| 17 | 0.291 | 0.130 |
| 18 | 0.113 | 0.145 |
| 19 | -0.231 | 0.094 |
| 20 | 0.043 | 0.345 |
| 21 | -0.163 | 0.034 |
| 22 | -0.260 | 0.061 |
| 23 | 0.053 | 0.001 |
| 24 | 0.190 | -0.082 |
| 25 | 0.236 | 0.466 |
| 26 | 0.029 | 0.071 |
| 27 | 0.240 | 0.192 |
| 28 | 0.051 | -0.156 |
| 29 | -0.075 | 0.277 |
| 30 | 0.133 | -0.171 |
